# ADAS-Cog Trajectories Differ from Expected Decline in Dementia Following Repeated Non-Invasive Interventions over 3 Years

**DOI:** 10.3390/medicina61111994

**Published:** 2025-11-06

**Authors:** Maria Anabel Uehara, Sumeet Kalia, Mari Garcia Campuzano, Zahra Moussavi

**Affiliations:** 1 Biomedical Engineering, University of Manitoba, Winnipeg, MB R3T 5V6, Canada; zahra.moussavi@umanitoba.ca; 2 Department of Statistics, University of Manitoba, Winnipeg, MB R3T 5V6, Canada; sumeet.kalia@umanitoba.ca; 3 Department of Electrical and Computer Engineering, University of Manitoba, Winnipeg, MB R3T 5V6, Canada; maritere.garciacampuzano@umanitoba.ca

**Keywords:** dementia, cognitive training, non-invasive brain stimulation, ADAS-Cog, washout, longitudinal

## Abstract

*Background and Objectives*: Non-pharmaceutical interventions such as cognitive training, transcranial electrical stimulation (tES), and repetitive transcranial magnetic stimulation (rTMS) have shown promise in improving cognitive outcomes in Alzheimer’s disease (AD) and dementia. However, the long-term effects of repeated non-invasive interventions remain unknown. This study investigated whether repeated non-invasive interventions administered over a span of 1 to 3 years were associated with slower cognitive decline compared to typical AD progression, and whether longer no-treatment intervals between treatments predicted greater post-treatment decline. *Materials and Methods*: Seventy-three participants living with dementia or AD received 2 to 9 blocks of non-invasive treatments (including tES, rTMS, cognitive training). Alzheimer’s Disease Assessment Scale-Cognitive subscale (ADAS-Cog) scores were collected longitudinally up to 3 years (36 months), across multiple intervention and assessment sessions. A mixed-effects model was used to estimate the rate of cognitive decline, adjusting for baseline age, sex, and baseline cognition (MoCA) with participants being the random effect. The observed rate of change was compared to a meta-analysis estimate of AD progression. Additionally, a linear mixed-effects model using robust sandwich estimation of standard errors was employed to assess whether the no-treatment interval was associated with changes in ADAS-Cog scores. *Results*: Participants showed a significantly slower rate of cognitive decline than expected from the AD reference rate (*p* < 0.001), with many demonstrating stabilized ADAS-Cog scores during their respective treatment periods, ranging from 1 to 3 years. Medication analyses revealed no significant effect of AD medications, antidepressants, antihypertensives, or cholesterol-lowering agents on cognitive outcomes. Furthermore, longer no-treatment intervals were significantly associated with greater post-treatment decline (*p* < 0.001). *Conclusions*: Repeated non-invasive treatments seem to slow the rate of cognitive decline in individuals living with dementia when administered over a prolonged period. This study provides evidence supporting the feasibility and effects of personalized long-term non-invasive treatment strategies for dementia.

## 1. Introduction

Over the last two decades, there have been thousands of studies analyzing the immediate and short-term effects of non-invasive interventions including cognitive training (CT) either alone or paired with electrical or magnetic stimulation in individuals with dementia and particularly with Alzheimer’s disease (AD). However, to our knowledge, no study to date has investigated the long-term effects of repeated blocks of non-invasive interventions, especially when administered over a period longer than one year, on sustained cognitive improvement in individuals living with dementia or AD.

Non-invasive brain stimulation (NIBS) interventions, including repetitive transcranial magnetic stimulation (rTMS) and transcranial electrical stimulation (tES) both aim to stimulate the brain without surgery. They use different mechanisms: rTMS uses pulsed magnetic fields whereas tES uses electric fields, but with commonality in that they try to modulate neuronal networks by either directly influencing neuron firing or by influencing excitability and plasticity [1].

There is growing evidence supporting the short-term and mid-term effects of non-invasive brain stimulation (NIBS) interventions, including repetitive transcranial magnetic stimulation (rTMS) and transcranial electrical stimulation (tES), in people living with AD/dementia. The two main types of tES are transcranial alternating current stimulation (tACS) and transcranial direct current stimulation (tDCS). A recent meta-analysis reported that anodal tDCS significantly improved global cognition in individuals with AD, compared to sham, as measured by the Mini-Mental State Examination (MMSE); they also demonstrated good tolerability, with only mild adverse effects and no difference in dropout rates between real and sham groups [2]. Protocols across the included studies ranged from single sessions to interventions lasting up to six months. Another meta-analysis similarly found that tDCS improved MMSE scores; in addition, they reported sustained effects lasting up to eight weeks [3]. Their analysis further indicated that stimulation sessions longer than 20 min and targeting the temporal cortex were more effective in enhancing global cognition than shorter sessions or left dorsolateral prefrontal cortex (DLPFC) stimulation [3]. In contrast, a separate meta-analysis found that both left DLPFC and temporal lobe stimulation enhanced memory performance, and that repeated tDCS sessions exerted cumulative positive effects on overall cognitive function, as assessed by multiple instruments including the Montreal Cognitive Assessment (MoCA), MMSE, and Alzheimer’s Disease Assessment Scale-Cognitive subscale (ADAS-Cog) [4].

While tDCS has been studied for many years, emerging evidence suggests that tACS may have similar or more potential to enhance cognitive function in individuals with AD. One systematic review found that gamma- and theta-tACS protocols were associated with improvements in memory, executive function, and global cognition, particularly in older adults with cognitive impairment [5]. However, the review also highlighted substantial methodological heterogeneity across studies—including differences in stimulation frequency, electrode montage, current intensity, session number, and cognitive outcome measures—which limits direct comparisons and generalizability. Another literature review focusing specifically on gamma-frequency tACS (typically 40 Hz) in mild cognitive impairment (MCI) and AD populations reported promising effects on memory and attention, with some studies suggesting that repeated sessions, many studies following 20 sessions over 4 weeks, may yield cumulative benefits [6]. These findings highlight the emerging potential of tACS for cognitive enhancement in AD, warranting further investigation into optimal stimulation parameters and long-term efficacy.

In addition to the literature on tES, one meta-analysis found that using high-frequency rTMS (≥10 Hz) targeting the DLPFC for people living with AD had significant improvement in cognitive outcomes such as ADAS-Cog and MMSE scores, immediately post-intervention compared to baseline [7]. This same meta-analysis found that significant results were from studies that had one block of treatment (including 20 or 30 successive treatment sessions) with excitatory stimulation (frequency ≥ 10 Hz) treatment sessions compared to those with a block of less than 10 sessions or those with inhibitory stimulation (rTMS frequency < 2 Hz). Furthermore, their analysis found that the observed improvements lasted about 1.5 months, with significantly better ADAS-Cog scores at the shorter follow-up durations compared to 3 months [7]. Another meta-analysis similarly reported significant improvement in global cognition, MMSE and MoCA, applying real rTMS treatment in AD and MCI populations at immediately post-treatment as well as at long-term (≥6 weeks) compared to those receiving sham stimulation [8]. The same meta-analysis study reviewed tDCS studies and found that tDCS improved memory and executive function but not global cognition [8].

While tES or rTMS alone has been shown to have short-term benefits, combining tES or rTMS with CT has been explored as a strategy for greater cognitive benefit [9,10,11]. A systematic review found that pairing rTMS or tDCS with CT significantly improved global cognition in people living with AD and MCI [9]. Subgroup analyses revealed that rTMS combined with CT was particularly effective, and participants with AD showed greater improvement compared to MCI. Additionally, tDCS combined with CT significantly improved language function but not global cognition or other cognitive domains. Follow-up assessment data ranged from 2 weeks to 6 months and indicated sustained benefits for global cognition and attention/working memory in individuals with AD receiving rTMS with CT but not tDCS with CT [9]. However, this result should be interpreted with caution, as the tDCS studies included only 3 to 24 sessions over 1 to 8 weeks, whereas the rTMS studies typically involved 20 to 30 sessions delivered over 4 weeks to 3 months. These findings suggest that combining rTMS or tDCS with CT may enhance cognitive outcomes more than either intervention alone, especially in AD population [9]. Evidence for stand-alone CT is, however, more muted. A review covering 11 studies in mild-to-moderate AD or vascular dementia found limited cognitive gains overall by CT interventions lasting between 5 and 24 weeks; though one high-quality trial of individualized cognitive rehabilitation did show promising improvements after 8 weeks of treatment in specific functional and memory tasks [10].

Several recent studies have begun to explore the effects of longer-term non-invasive interventions in individuals living with AD, highlighting the potential benefits of sustained treatment over extended periods. For example, our research team has previously run a randomized crossover sham-controlled clinical trial on the efficacy of 40 Hz tACS paired with CT on dementia [11]. This crossover design had two 4-week treatment blocks (either real tACS or sham) separated by a minimum of 8 weeks. We found that the group who received real tACS first had sustained cognitive improvement during the washout period of on average 11 weeks compared to the group that received sham first [11]. Furthermore, the overall trend for all participants was that they improved by the end of the study compared to their baseline score. One study conducted 20 Hz-rTMS over the precuneus over 52 weeks in people living with mild-to-moderate AD and found that the real rTMS experienced significantly lower cognitive decline, using Clinical Dementia Rating Sum of Boxes and ADAS-Cog, preserved daily functioning, and reduced behavioral symptoms compared to sham [12]. This intervention involved 2 weeks of treatments 5 days per week and then one treatment a week for 50 weeks [12]. Similarly, another study conducted the largest multisite randomized controlled rTMS clinical trial following participants for up to 6 months post-treatment [13]. Both active and sham groups showed sustained cognitive improvement up to 2 months post-treatment [13]. The intervention involved 2 or 4 weeks of daily rTMS over bilateral DLPFC, with follow-up assessments extending to 28 weeks post-baseline [13]. Another randomized controlled trial evaluated the effects of repeated cycles of CT over one year in participants living with AD receiving cholinesterase inhibitors. The experimental group underwent five 4-week blocks of CT (20 sessions per block, 2 h/day, 5 days/week) with a 4-week break between treatment blocks [14]. Compared to controls who received non-specific cognitive activities, the experimental group showed significant improvement in MMSE, Milan Overall Dementia Assessment, and multiple neuropsychological tests compared to controls receiving non-specific cognitive activities [14]. Even with these findings from one-year interventions, the literature still lacks investigations of long-term, repeated, multi-modal non-invasive interventions, especially those beyond one year and including multiple treatment blocks.

It has been hypothesized that repeated or maintenance stimulation over extended periods may sustain and strengthen neuroplastic changes induced by the treatment. Studies have suggested that repeated NIBS can lead to lasting changes caused by synaptic long-term potentiation (LTP), cortical excitability, and changes in functional and structural connectivity of networks [1,15]. In dementia, repeated stimulation and cognitive engagement may help maintain neural circuits and slowing decline. Previous studies have proposed that maintenance or booster stimulation sessions could prolong the treatment effects [12,16,17]. This supports the rationale for investigating the long-term effects of repeated non-invasive interventions over time.

Our team has been investigating the efficacy of various non-invasive interventions as a treatment for individuals living with AD/dementia repeatedly over the past 7 years; thus, we have cognitive assessment data of the same patients over the years. In this study, we aimed to analyze longitudinal data from participants who received at least two rounds of non-invasive treatment, including tACS, tDCS, rTMS with or without CT and only CT intervention over a span of 3 years. As individuals with neurodegenerative dementia are expected to decline over time, we hypothesize there would be a significantly slower rate of decline for those receiving repeated NIBS and/or CT compared to untreated patients. We also explored whether dementia-related medication would have any effect on the rate of decline, and also whether longer treatment gaps would be associated with greater post-treatment cognitive decline. To our knowledge, this is the first study of its kind to integrate longitudinal cognitive data, no-treatment interval analysis, and the impact of medication in evaluating the long-term effects of repeated non-invasive interventions. Determining whether repeated non-invasive interventions can slow cognitive decline is critical, as it would demonstrate their potential as a long-term treatment strategy to help maintain independence, reduce caregiver burden, delay institutionalization, lower healthcare costs, and improve quality of life for individuals living with dementia.

## 2. Materials and Methods

### 2.1. Participants

**Figure 1:**
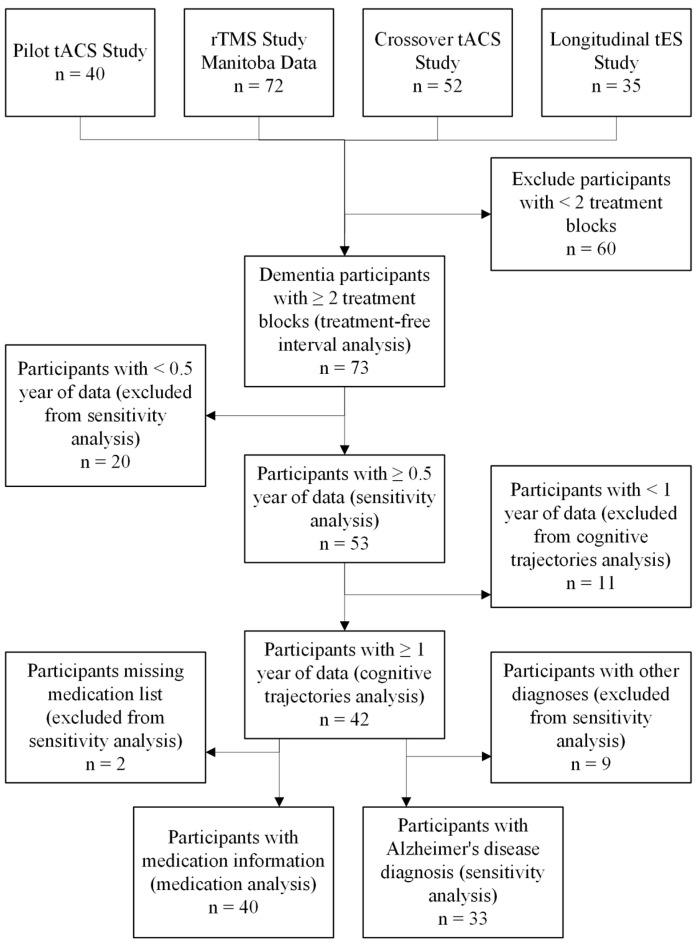
Flowchart of participants who received at least two treatment blocks and analysis inclusion.

Participants were enrolled in a series of non-invasive interventions involving tES [11,18,19], rTMS [13], and/or CT. This analysis included 73 participants with dementia who received between 2 and 9 treatment blocks; each block was 20 sessions over 4 weeks with the exception of one block of treatment that was 10 sessions over two-weeks of rTMS treatment (*n* = 7) and one block of treatment that was 24 sessions over 8 weeks tACS paired with CT treatment (*n* = 2). Participants were recruited through posters posted throughout the community and physicians’ referrals, and all clinical trials were conducted at Riverview Health Centre in Winnipeg, MB, Canada. Figure 1 shows a flowchart of the above-mentioned studies and inclusion of the participants. Table 1 summarizes participant flow for each source study, including total enrollment, number who completed the study, number who withdrew, and number whose data were included in this analysis. For the first three studies [11,13,18,19], all participants included in this analysis completed the study, and there were no withdrawals. For the first study [18,19], withdrawals occurred either due to the COVID-19 pandemic or participants choosing not to continue after completing a treatment block. For the second study [13], reasons for withdrawal are detailed in [20] with a full analysis of attrition. For the third study [11], withdrawal reasons are summarized in Table 1 of the original publication. For the fourth study [21], it is ongoing and thus, most participants have not yet completed the study which involves multiple treatment blocks with washout periods in between. The analysis for this study used the data available up to 3 September 2025. All clinical trials received ethics approval from the University of Manitoba Biomedical Research Ethics Board and were registered on ClinicalTrials.gov site online. Informed consent was obtained from all participants prior to their enrollment in each of the original studies included in this analysis.

### 2.2. Study Design

The studies from which we adopted data varied in design and intervention structure. In the pilot study, participants were assigned to either CT alone for 4 weeks or CT paired with tACS delivered for 4 or 8 weeks [18,19]. A randomized controlled trial compared two active rTMS protocols (2 or 4 weeks) against sham stimulation [13]. Another study used a crossover design in which participants completed both real and sham tACS combined with CT, separated by an average washout period of 11 weeks [11]. The ongoing longitudinal study enrolls participants in a single-arm protocol where each participant receives multiple tES modalities in randomized order, with washout period of 2–5 months between blocks [21]. Table 2 includes summarized information about the different interventions.

### 2.3. Treatment Protocols

Despite differences in design, the interventions shared several commonalities. First, the stimulation target across all brain stimulation protocols was the DLPFC. For tACS and tDCS protocols, the reference electrode was placed over the right supraorbital area. Second, CT was consistently delivered using the MindTriggers app (version 1.0.0 to 3.0.0) with support from a treatment administrator. Third, assessments were conducted before and after each treatment block, with some protocols also including 1-month follow-up assessments; the rTMS study [13] had four follow-up assessments up to 6 months post-treatment.

As can be seen in Table 2, treatment blocks consisted of one of the following protocols: gamma tACS combined with CT, theta tACS with CT, tDCS with CT, real rTMS, sham rTMS, sham tES with CT, or CT alone. Each treatment block consisted of 20 sessions administered on weekdays over 4 weeks, with the exception of 2 participants who completed the 8 weeks (3 days/week, 24 total sessions) of tACS + CT and 7 participants who completed 2 weeks (5 days/week, 10 total sessions) of rTMS. Participants received the same protocol per treatment block and treatments generally lasted between 20 min and 1.5 h, often involving two 30 min treatments separated by a break. Detailed methodologies for each intervention can be found in [19,21,22,23].

### 2.4. Inclusion and Exclusion Criteria

Inclusion criteria across studies typically required a physician-confirmed diagnosis of dementia. However, the rTMS study specifically required a diagnosis of AD. Additional common criteria included the ability to read and write in English or Spanish to complete assessments, and a MoCA score below 26. Participants were excluded if they had a history of epileptic seizures or epilepsy (for brain stimulation protocols), uncorrected visual or hearing impairment that interfered with cognitive testing, substance use disorder, participating in another therapeutic study for dementia, change in dementia-related medication during the treatment block, or a major mood disorder diagnosis.

For the current analysis, participants were eligible if they had completed at least two non-invasive interventions as part of the original studies. To ensure sufficient longitudinal data, only individuals with cognitive assessments (ADAS-Cog or Wechsler Memory Scale) spanning 1 to 3 years were included in the main analysis. These data could originate from a single study or multiple studies among the four studies’ datasets. Participants with less than 6 months of data were excluded from the dataset.

### 2.5. Outcome Measures

The primary outcome was the ADAS-Cog [24] or completed Wechsler Memory Scale (WMS) [25] in some of the studies. For ADAS-Cog, Scores range from 0 to 70, with higher scores indicating greater cognitive impairment. The ADAS-Cog includes tasks assessing memory, language, praxis, and orientation. In the pilot tACS study [18,19], WMS was administered in place of ADAS-Cog as can be seen in Table 2. Therefore, the data from that pilot study required conversion using a predictive model to enable longitudinal integration with the studies using ADAS-Cog.

The assessments were administered at variable intervals: at baseline, post-treatment, and (for most studies) at follow-up. To standardize timepoints across participants, we created a six-month grid (0, 6, 12, 18, 24, and 30 months) and assigned the closest available assessment to each timepoint, allowing a maximum deviation of one month. Some participants (*n* = 7) had long periods (up to 6 months) between treatment blocks with no assessments; thus, we linearly interpolated the ADAS-Cog score with the assessments around the timepoint of interest. The average deviation from exact 6-month spacing was less than one month.

### 2.6. Estimating ADAS-Cog from WMS Data

To be able to compare data from all these studies, some studies used only WMS as the outcome measure while the majority of them used ADAS-Cog or both ADAS-Cog and WMS. Thus, we had to convert one scale to another in order to compare the cognitive status of participants over time. To estimate ADAS-Cog scores from WMS subcomponents, a multiple linear regression model was fitted using all available paired longitudinal assessments (*n* = 378) from 57 participants who had completed both WMS and ADAS-Cog within the same week. Twenty WMS subcomponents were included as predictors, including Logical Memory, Visual Reproduction, Verbal Paired Associates, Immediate and Delayed Memory Indices, and composite memory scores. While the ADAS-Cog distribution exhibited mild positive skewness and heteroscedasticity, model residuals were approximately symmetric, and prediction performance was stable. To account for potential non-constant variance, robust (heteroscedasticity-consistent) standard errors were used. Model performance was evaluated using the root mean square error, mean absolute error, and intraclass correlation coefficient. The final model was applied to 111 assessments with only WMS from 26 participants to estimate ADAS-Cog scores. The decision to estimate ADAS-Cog from WMS was based on the fact that both assess cognitive function, particularly memory, with several overlapping components. The decision to estimate ADAS-Cog from WMS is supported by prior studies demonstrating strong correlations between the two measures [26] and high test–retest reliability of WMS-IV in dementia populations [27].

### 2.7. Cognitive Trajectories Analysis

To assess whether repeated non-invasive treatments were associated with a slower rate of cognitive decline over time, we used a linear mixed effects model with ADAS-Cog as the outcome and time (in months) as a fixed effect. The model included fixed effects for baseline age, sex, and baseline MoCA score as covariates. A random intercept was specified for each participant to account for within-subject correlation due to repeated measures. The model was estimated using the lme4 (version 1.1-37) and lmerTest (version 3.1-3) packages in R version 4.3.2 [28].

The primary analysis included participants who received at least two treatment blocks and had at least 1 year of data (*n* = 42) with various durations: 19 participants had data up to 1 year, 5 up to 1.5 years, 5 up to 2 years, 6 up to 2.5 years, and 7 up to 3 years. Model assumptions were evaluated via visual inspection of residuals, and multicollinearity was assessed using variance inflation factors, all of which were below a threshold of 5.

To compare the observed rate of ADAS-Cog change in our studies with the average rate of progression reported in a meta-analysis of 140 clinical trials, which estimated a mean decline of 5.82 points per year (0.485 points/month) in placebo-treated participants [29], we conducted a one sample *t*-test. The statistic was calculated as the difference between the estimated slope and the reference value, divided by the standard error of the estimated slope. Because the one-sample *t*-test assumes independent and identically distributed observations, we used robust sandwich estimator of standard errors to account for potential correlation among repeated measures. Robust standard errors were estimated using the sandwich package (version 3.1-1) in R [30].

Two sensitivity analyses were conducted to assess the robustness of the primary findings. Firstly, we repeated the model using all available data, including participants with only 6 months of assessments (*n* = 53). The second analysis repeated the model using all available data with restricted samples to participants diagnosed with AD.

### 2.8. Medication Analysis

To examine whether concurrent medications influenced cognitive trajectories, we conducted exploratory analyses by medication category. Medication lists were recorded at baseline of each treatment block, and participants did not change medication or dosage during the treatment and follow-up assessments. It was also confirmed that participants did not have medication changes between different treatment blocks. Approximately half of the total sample was included in these medication analyses, and this subgroup was drawn from the primary analysis dataset. Participants were grouped dichotomously for each medication category:

- AD medications: cholinesterase inhibitors (donepezil, galantamine, rivastigmine) or memantine
- Antidepressants: selective serotonin reuptake inhibitors (SSRIs) (paroxetine, citalopram, escitalopram, fluoxetine, sertraline) or other types (bupropion, trazodone)
- Hypertension medications: valsartan, telmisartan, ramipril, enalapril, candesartan, irbesartan, perindopril, fosinopril, hydrochlorothiazide, nifedipine, amlodipine, bisoprolol, atenolol, metoprolol
- Cholesterol medications: atorvastatin, rosuvastatin, simvastatin, ezetimibe

For each medication category, we fit a linear mixed-effects model with ADAS-Cog as the outcome. Fixed effects included time (in months), medication status, and their interaction, along with baseline age, sex, and MoCA score as covariates. The interaction term tested whether the rate of cognitive change differed by medication group. Random intercepts were specified for each participant to account for repeated measures.

### 2.9. No-Treatment Interval Analysis

To investigate the effect of no-treatment intervals on changes in cognitive function, we employed a robust linear mixed-effects model using the rlmer function from the R package robustlmm (version 3.3-3) [28]. The dependent variable was the change in ADAS-Cog score, and the primary predictor was the duration of the no-treatment interval (days). Participant-level random intercepts accounted for repeated measures. Robust sandwich estimation was applied to address deviations from normality and heteroscedasticity [31]. This analysis included all 73 participants, contributing 266 observations. No-treatment intervals ranged from 3 weeks to 5.5 years. These intervals include intervals between treatment blocks within a study, gaps between separate studies, or the time from the last treatment to a follow-up assessment.

## 3. Results

Participant demographics for the ADAS-Cog trajectories analysis (*n* = 42) are shown in Table 3, including a breakdown of clinical diagnoses. Participants received between 2 and 9 blocks of non-invasive treatment and were followed for up to 3 years. The number of participants contributing data up to each timepoint was: 19 at 1 year, 5 at 1.5 years, 5 at 2 years, 6 at 2.5 years, and 7 at 3 years.

### 3.1. Estimating ADAS-Cog from WMS Data

To compare across studies that used WMS and ADAS-Cog, we developed a method to estimate ADAS-Cog scores for assessments where only the WMS was available. A multiple linear regression model was fitted using all available paired longitudinal data (378 assessments from 57 participants). The model achieved good predictive performance with a root mean square error of 4.92 and a mean absolute error of 3.60. Agreement between observed and predicted ADAS-Cog values was high, as indicated by an intraclass correlation coefficient of 0.86 (95% CI: 0.83 to 0.88, *p* < 0.001). Model residuals showed mild deviation from normality (Shapiro–Wilk *p* < 0.001) and heteroscedasticity (Breusch–Pagan *p* < 0.001); thus, robust standard errors were used. The estimated coefficients and corresponding robust *p*-values are shown in Appendix A, Table A1.

### 3.2. Cognitive Trajectories Analysis

A linear mixed effects model was used to assess longitudinal changes in ADAS-Cog scores among participants who received at least two blocks of non-invasive treatment and completed at least three assessments (*n* = 42). The model included fixed effects for time (in months), baseline age, sex, and baseline MoCA score, with a random intercept for participant. Time was modeled as a continuous linear variable. There was a significant effect of time in months (β = 0.23, *p* < 0.001), indicating that ADAS-Cog scores increased gradually over the assessed period but at a slower rate than typically expected. Higher baseline MoCA scores were significantly associated with lower ADAS-Cog scores (β = −1.48, *p* < 0.001). Age (β = 0.06, *p* = 0.56) and sex (β = −1.05, *p* = 0.54) were not significant predictors. The random intercept standard deviation was 4.50, and the residual standard deviation was 5.66. The model explained 53% of the variance in ADAS-Cog scores attributable to fixed effects and 71% when including between-subject variability. The full fixed-effect results can be found in Appendix B, Table A2. Model diagnostics confirmed that residuals were normally distributed with no major deviations or heteroscedasticity. Variance inflation factors for all predictors were below 2, indicating no multicollinearity.

**Figure 2:**
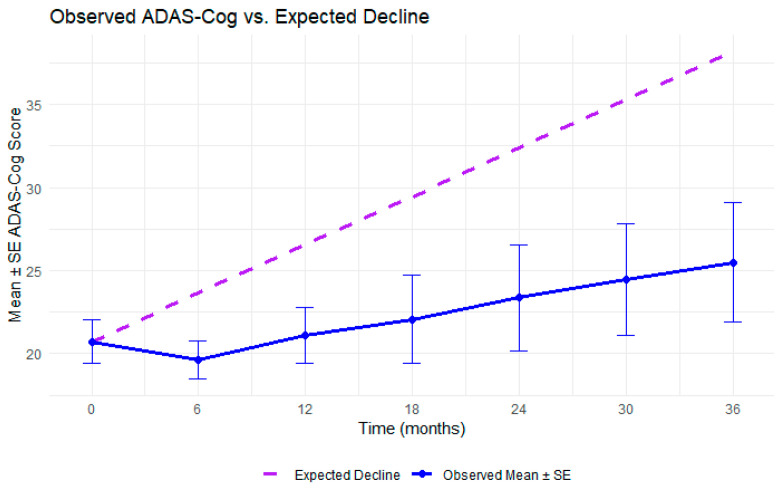
Overall trajectory of the observed ADAS-Cog scores for participants who received multiple treatment blocks compared to the expected decline in AD.

**Figure 3:**
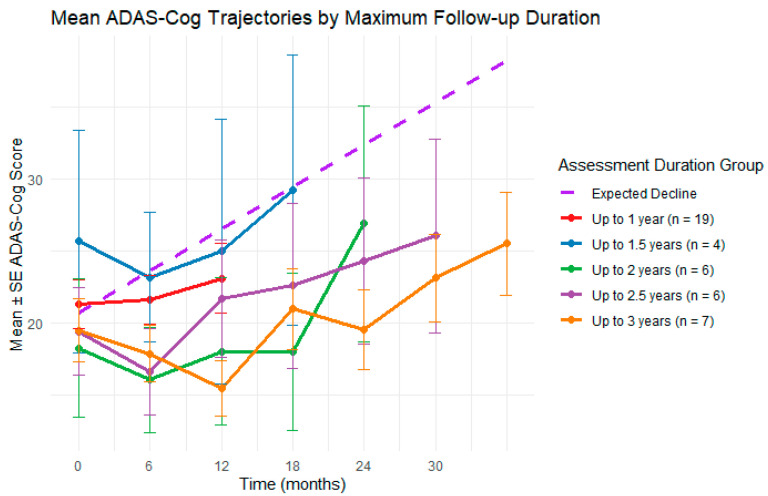
Observed ADAS-Cog trajectories stratified by the maximum duration of available assessment data, rounded down to 6-month intervals, ranging from 1 year to 3 years. Assessments were associated with repeated treatment blocks, with participants receiving between 2 and 9 treatment blocks during these periods.

To contextualize the observed slope, we conducted a one-sample *t*-test comparing the rate of ADAS-Cog change in this cohort to the average AD progression rate reported in a meta-analysis of 140 clinical trials (0.485 points/month) [29]. Robust standard errors were used to account for potential violations of independence assumption inherent to repeated measures data. The observed slope (0.23 ± 0.07, 95% CI: 0.09 to 0.37) was significantly lower than the expected rate (t = −3.59, df = 163.04, *p* < 0.001), suggesting that participants in this study experienced slower-than-expected cognitive decline. Figure 2 shows the observed ADAS-Cog trajectory alongside the expected decline in untreated AD. Figure 3 shows a similar plot with observed ADAS-Cog scores stratified by maximum assessment duration.

As a sensitivity analysis, we repeated the model using all participants who received at least two treatment blocks, with at least 6 months of data (*n* = 53). Results were consistent with the primary analysis: both the effect of time in months (β = 0.19, *p* < 0.001), and the MoCA score (β = −1.61, *p* < 0.001) remained a strong predictor, supporting the robustness of the findings. The full fixed-effect results can be found in Appendix B, Table A3. We also conducted a second sensitivity analysis restricted to participants diagnosed with AD (*n* = 36). The effect of time remained significant (β = 0.20, *p* < 0.001), and MoCA score continued to be a strong negative predictor of ADAS-Cog performance (β = −1.45, *p* < 0.001), further supporting the consistency of the observed trajectories across subgroups. The full-fixed effect results can be found in Appendix B, Table A4.

### 3.3. Medication Analysis

The exploratory analysis on whether medication modified cognitive trajectories across all models showed that baseline MoCA scores and time (in months) remained strong predictors of ADAS-Cog. However, none of the medication categories significantly influenced baseline ADAS-Cog or the rate of cognitive change. For AD medications, neither the main effect (*p* = 0.12) nor the interaction with time (*p* = 0.30) reached significance. Similarly for antidepressants (main effect: *p* = 0.11; interaction: *p* = 0.78), hypertension medications (main effect: *p* = 0.35; interaction: = 0.40), and cholesterol medication (main effect: *p* = 0.83; interaction: *p* = 0.23) showed no significant associations with ADAS-Cog scores. These findings suggest that within this sample, use of common AD, psychiatric, or cardiovascular medications did not alter the trajectory of cognitive change during repeated non-invasive interventions. All model full fixed-effect results can be found in Appendix B, Table A5, Table A6, Table A7 and Table A8.

### 3.4. No-Treatment Interval Analysis

**Figure 4:**
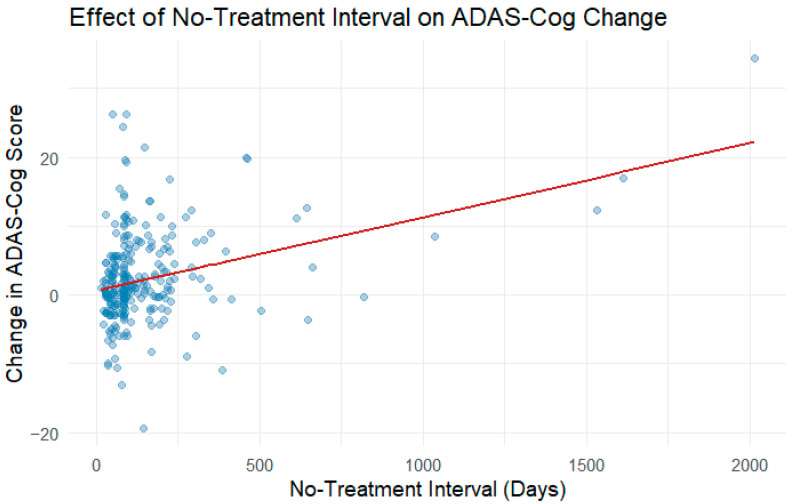
Association between no-treatment interval and change in ADAS-Cog score. Each point represents an observation from an individual participant. The solid red line indicates the fitted relationship from the robust mixed-effects model, showing an overall positive association between longer no-treatment intervals and greater cognitive decline.

Participant demographics for the no-treatment interval analysis (*n* = 73) are shown in Table 2, including a breakdown of clinical diagnoses. The robust mixed model revealed a statistically significant positive relationship between no-treatment interval and cognitive decline, with an estimated increase in ADAS-Cog change of 0.0108 points per additional no-treatment day (95% CI not reported here; SE = 0.002; t = 6.892, *p* < 0.001). This suggests that longer intervals without treatment are associated with cognitive decline (Figure 4). The estimated intercept, representing the predicted cognitive change immediately after treatment, was positive but not statistically significant (Estimate = 0.556, SE = 0.404, t = 1.375). Between-subject variability in baseline cognitive change was minimal after accounting for no-treatment days, as indicated by negligible random intercept variance. Robustness weights showed that a minority of residuals were down weighted, confirming the presence of some outliers that were controlled in the analysis.

## 4. Discussion

This study investigated the long-term ADAS-Cog trajectory of participants with dementia or AD who received multiple rounds of non-invasive treatments, including tACS, tDCS, rTMS, and CT, over a period of 1 to 3 years. By tracking ADAS-Cog scores at multiple timepoints, we estimated the rate of decline over time and compared it to the expected trajectory of untreated AD. A meta-analysis on AD of over 140 clinical trials reports an average decline of 5.82 ADAS-Cog points per year (approximately 0.485 points per month) in placebo-treated participants, which serves as our reference rate of decline [29]. Our participants had a mean age of 72.1 years and baseline ADAS-Cog of 20.7, compared to 73.5 years and 24.5 ADAS-Cog score in the meta-analysis. These differences were modest, particularly for age, supporting the validity of this comparison, but it is important to note the slightly milder baseline impairment in our sample. The results showed that the rate of cognitive decline, as measured by the ADAS-Cog, was significantly slower than the average decline in AD [29]. In fact, for many participants, cognitive scores remained stable over time, diverging meaningfully from the expected trajectory of disease progression.

This study adds to the growing research largely focused on short-term outcomes. The meta-analyses of high-frequency rTMS (>5 Hz) over the DLPFC have shown improvements in ADAS-Cog, MMSE, and MoCA, but the longest follow-up in these studies was only three months, with most follow-ups under 6 months [7,8]. Similarly, combining tDCS with CT has been shown to produce short-term gains in working memory, attention, or language function, and stand-alone CT programs has shown modest benefits [10] with diminishing effects within months. Even the long-term studies, such as 52-week rTMS over the precuneus [12] or 12-month CT programs [14,32], have not explored repeated treatment blocks or follow-up beyond a year.

Most importantly, the observed slowing of cognitive decline may have a higher significance beyond the test scores. Because transportation was confirmed with each participant for every treatment block, we know that none of our participants were institutionalized during the assessments of this study. These outcomes, though they are difficult to record in a standardized method, indicate the potential of repeated, long-term non-invasive interventions not only to slow measurable cognitive decline but also to preserve autonomy and reduce caregiver burden. These functional implications align with the benefits in daily functioning reported in the year-long rTMS and multimodal CT trials [14,32] and strengthen the argument for sustained, maintenance-oriented intervention strategies in AD.

The secondary analysis revealed a significant association between longer no-treatment intervals and increased cognitive decline, with ADAS-Cog scores worsening by approximately 0.01 per additional day without intervention. This finding emphasizes the importance of consistent and repeated treatment periods in maintaining cognitive function. While most studies focus on the efficacy of the intervention and its sustained effects [7,8,9,10], or a prolonged treatment protocol such as weekly treatments for a year [12,14,32], our results suggest that a balance between the cognitive decline during a no-treatment interval and accommodating personal schedule requires further research. Prior studies found that discontinuation of medication such as cholinesterase inhibitors in AD can lead to cognitive decline [33,34]. Many participants’ caregivers expressed a preference for treatment blocks spaced out over time to accommodate personal schedules, including travel, seasonal accessibility, and lifestyle flexibility. These preferences highlight the need for adaptable treatment models that balance clinical efficacy with real-world feasibility. Our findings support the possibility of delivering interventions in periodic blocks, provided that washout durations are carefully managed to minimize cognitive regression. Future studies should explore optimal scheduling strategies, including the maximum tolerable washout length, to guide maintenance protocols that preserve cognitive stability while respecting individual needs.

Analyses of medication use revealed that AD medications, antidepressants, hypertension medications, and cholesterol medications had no significant effect on the rate of cognitive decline. All participants were required to be on stable doses of medication prior to and throughout the study period, and we verified that no changes in medications occurred between different studies for those who were part of multiple studies. Thus, the educed rate of cognitive decline in our study population was unlikely to be attributable to pharmacological treatment. However, subgroup sizes were limited, particularly for antidepressants, so these results should be interpreted with caution. Although our study found no significant effects of medications on cognitive trajectory, previous research suggests that certain medication classes such as SSRIs, cholinesterase inhibitors, and N-methyl-D-aspartate receptor antagonists may modulate the effects of tDCS [35,36,37,38]. However, evidence for similar interactions with rTMS and tACS remains limited.

There are several strengths in this study. First, to the best of our knowledge, this is the longest longitudinal assessment period in a population receiving non-invasive interventions for dementia, with some participants followed for over 3 years. Second, the use of linear mixed models with participant-level random intercepts allowed us to estimate the average rate of cognitive change over time while accounting for individual variability. Third, by comparing the observed rate of change to the average decline rate derived from a meta-analysis of 140 placebo-controlled AD clinical trials, we were able to compare our results to the expected decline rate. This comparison enhances the clinical relevance of our findings and provides a clearer interpretation than relying solely on within-group change scores. Finally, the sensitivity analyses, including models that use all available data and those restricted to participants diagnosed with AD, yielded consistent results, further supporting the robustness of our findings.

There are several limitations including no untreated or placebo control group within our dataset. While the comparison to an external benchmark offers a meaningful reference, a more rigorous approach would involve a randomized control condition to ensure baseline characteristics, such as population characteristics, severity, and assessment protocols, are comparable across groups. Second, participants received different types of non-invasive treatments from different studies, complicating the interpretation of which treatments were the most effective or if receiving more than one type of treatment is better. While this heterogeneity reflects real-world clinical practice, where treatment is often tailored to individual needs and disease progression, it also introduced variability in study design and follow-up schedules. Although combining the datasets was justified by the shared outcome measure (ADAS-Cog) and using standardized timepoints, this heterogeneity complicates interpretation and limits generalizability. Third, conversion of WMS scores to ADAS-Cog introduces potential measurement error, and assuming linear change over time may oversimplify the trajectory of cognitive decline. Fourth, the sample size at longer follow-up intervals was limited which may introduce bias or reduce power to detect small effects. Additionally, the modest sample sizes within multiple subgroup analyses may further limit statistical power. Fifth, because inclusion required at least one year of follow-up and participants who continued receiving multiple treatments were retained, introducing selection bias, limiting generalizability to more advanced individuals or higher dropout risk. Finally, in a small number of cases (7 out of 231 data entries), we linearly interpolated around the regularly scheduled timepoint to retain longitudinal continuity. However, cognitive decline in dementia is rarely linear, and this approach may artificially smooth or underestimate decline.

## 5. Conclusions

Overall, our findings suggest that repeated, personalized, non-invasive interventions may help sustain cognitive function and slow the decline of the disease in individuals with dementia over time. These results question the current understanding that such treatments only produce immediate or short-term effects and provide initial evidence that prolonged use may offer sustained benefit. A periodic “booster” treatment of NIBS and/or CT may preserve cognition over many years. Future research should systematically evaluate optimal treatment schedules, combinations of modalities, and patient characteristics that predict long-term responsiveness. By reframing intervention strategies in AD to include ongoing, low-burden, non-pharmacological maintenance, it may be possible to meaningfully extend independence and quality of life for people living with dementia.

## Figures and Tables

**Table 1:** Summary of participant enrollment, completion, withdrawal, and inclusion in analysis for each study.

Study	Total Enrolled	Completed	Withdrawn	Used in Analysis
[18,19]	*n* = 40	*n* = 31	*n* = 9	*n* = 26
[13]	*n* = 72	*n* = 61	*n* = 11	*n* = 33
[11]	*n* = 52	*n* = 42	*n* = 10	*n* = 42
[21]	*n* = 35	*n* = 8	*n* = 6	*n* = 28

**Table 2:** Summary of included clinical trials contributing data to the current longitudinal analysis.

Study	Study Design	Assessments	Treatment Schedule	Stimulation Parameters
[18,19]	3 groups, no randomization, no blinding: CT only (4 weeks) tACS + CT (4 weeks) tACS + CT (8 weeks)	Baseline, post-treatment, 1-month follow-up: WMS MADRS	5 days/week, 4 weeks OR 3 days/week, 8 weeks	Two 30 min sessions/day with a 30-min break in between, 40 Hz tACS, 0.75 mA, left DLPFC
[13,22]	3 groups, stratified randomization, double-blind: Active rTMS (2 weeks) Active rTMS (4 weeks) Sham rTMS (4 weeks)	Baseline (week 0), week 3, 5, 12, 20 and 28: ADAS-Cog	5 days/week for 2 or 4 weeks	20 Hz, 1.5 s trains with 10 s intertrain interval, 25 trains bilaterally, 90–100% resting motor threshold, left and right DLPFC
[11]	Crossover, stratified randomization, double-blind: tACS + CT (4 weeks), washout (2–7 months), sham + CT (4 weeks) sham + CT (4 weeks), washout (2–7 months), tACS + CT (4 weeks)	Baseline, post-treatment: ADAS-Cog WMS (missing data) MADRS	Two 30 min sessions/day, 5 days/week for 4 weeks, with average washout period of 11 weeks	Two 30 min sessions/day with a 30-min break in between, 40 Hz tACS, 0.75 mA, left DLPFC
[21]	Single-arm, simple randomization, double-blind, longitudinal, each participant receives all tES protocols + CT in randomized order for 4 weeks with a 2–5-month washout period	Baseline, post-treatment, 1-month follow-up: ADAS-Cog WMS (missing data) MADRS	Two 30 min sessions/day, 5 days/week for 4 weeks, with washout period between treatment blocks of 2–5 months	Two 30 min sessions/day with a 30 min break in between, left DLPFC Personalized gamma-tACS (0.75 mA) Personalized theta-tACS (1 mA) tDCS (1 mA) Sham tACS

Abbreviations: ADAS-Cog = Alzheimer’s Disease Assessment Scale—Cognitive Subscale, CT = cognitive training, DLPFC = dorsolateral prefrontal cortex, MADRS = Montgomery–Åsberg Depression Rating Scale, rTMS = repetitive transcranial magnetic stimulation, tACS = transcranial alternating current stimulation, tDCS = transcranial direct current stimulation, tES = transcranial electrical stimulation, WMS = Weschler Memory Scale.

**Table 3:** Participant demographic and clinical characteristics at baseline by analysis (cognitive trajectories and no-treatment interval).

Characteristic	Categories	Cognitive Trajectories Analysis		No-Treatment Interval Analysis	
		Count (*n*)	Percent (%)	Count (*n*)	Percent (%)
Sex	Female	17	40.5	26	35.6
	Male	25	59.5	47	64.4
Education	Primary education	8	19	12	16.4
	Secondary education	10	23.8	18	24.7
	Some post-secondary education	3	7.1	6	8.2
	College Diploma	2	4.8	3	4.1
	Bachelor’s degree	11	26.2	20	27.4
	Master’s degree	5	11.9	8	11.0
	Doctorate degree	3	7.1	6	8.2
Handedness	Right	38	90.5	67	91.8
	Left	3	7.1	5	6.8
	Ambidextrous	1	2.4	1	1.4
Diagnosis	Alzheimer’s disease	32	78	46	67.7
	Dementia	7	14.7	12	16.4
	Vascular Dementia	2	4.9	7	9.6
	Mild Cognitive Impairment	1	2.4	5	6.8
	Posterior Cortical Atrophy	0	0	1	1.4
	Lewy Body Dementia	0	0	1	1.4
	Frontotemporal Dementia	0	0	1	1.4
Medication	Alzheimer’s Disease	23	57.5		
	Antidepressants	7	17.5		
	Hypertension	17	55		
	Cholesterol	18	45		
		Mean	SD	Mean	SD
Age (years)		72.1	8.9	73.6	8.6
MoCA		17.1	5.1	17.1	5.3
MADRS		6.1	8.1	5.3	6.6
ADAS-Cog		20.7	8.6	21.7	9.2

Abbreviations: ADAS-Cog = Alzheimer’s Disease Assessment Scale—Cognitive Subscale, MADRS = Montgomery–Åsberg Depression Rating Scale, MoCA = Montreal Cognitive Assessment.

## Data Availability

The data that support the findings of this study are available from the PI of the studies (Author Z.M.) upon request.

## Abbreviations

The following abbreviations are used in this manuscript:

AD	Alzheimer’s Disease
ADAS-Cog	Alzheimer’s Disease Assessment Scale–Cognitive Subscale
CT	Cognitive Training
DLPFC	Dorsolateral Prefrontal Cortex
LASSO	Least Absolute Shrinkage and Selection Operator
MADRS	Montgomery–Asberg Depression Rating Scale
MCI	Mild Cognitive Impairment
MMSE	Mini-Mental State Examination
MoCA	Montreal Cognitive Assessment
NIBS	Non-Invasive Brain Stimulation
rTMS	Repetitive Transcranial Magnetic Stimulation
SSRI	Selective Serotonin Reuptake Inhibitor
tACS	Transcranial Alternating Current Stimulation
tDCS	Transcranial Direct Current Stimulation
tES	Transcranial Electrical Stimulation
WMS	Wechsler Memory Scale

## Appendix A

**Table A1:** Multiple linear regression coefficients for predicting ADAS-Cog from WMS components.

Variable	Coefficient (β)	Robust SE	t Value	*p* Value
Intercept	55.032	10.212	5.389	1.29 × 10^−7^
Brief Cognitive Status Exam Total Raw Score	−0.222	0.033	−6.716	7.38 × 10^−11^
Logical Memory I	−0.659	0.155	−4.251	2.72 × 10^−5^
Logical Memory II	0.251	0.145	1.733	0.084
Verbal Paired Associates I	−0.682	0.190	−3.599	3.65 × 10^−4^
Verbal Paired Associates II	0.692	0.491	1.410	0.159
Visual Reproduction I	−0.152	0.144	−1.053	0.293
Visual Reproduction II	0.461	0.176	2.616	0.009
Symbol Span	−0.081	0.062	−1.312	0.190
Logical Memory II Recognition	0.116	0.089	1.308	0.192
Verbal Paired Associates II Recognition	−0.174	0.063	−2.750	0.006
Verbal Paired Associates II Recall	0.079	0.096	0.820	0.413
Visual Reproduction II Recognition	0.010	0.233	0.042	0.967
Auditory Memory Sum of Scaled Scores	−0.562	0.677	−0.831	0.407
Auditory Memory Index	−0.766	0.341	−2.245	0.025
Visual Memory Sum of Scaled Scores	−1.760	0.794	−2.217	0.027
Visual Memory Index	−0.283	0.142	−2.001	0.046
Immediate Memory Sum of Scaled Scores	2.920	0.866	3.372	8.28 × 10^−4^
Immediate Memory Index	0.040	0.042	0.950	0.343
Delayed Memory Sum of Scaled Scores	0.614	0.386	1.591	0.112

## Appendix B

**Table A2:** Fixed effects from the linear mixed-effects model estimating longitudinal changes in ADAS-Cog scores (*n* = 42). Estimates are adjusted for baseline age, sex, and MoCA score. A random intercept was included for each participant.

	Estimate	SE	df	t Value	*p* Value
Intercept	41.249	7.254	38.085	5.686	1.52 × 10^−6^
Time (months)	0.227	0.045	163.040	5.089	9.82 × 10^−7^
Baseline Age (years)	0.056	0.095	39.322	0.591	0.558
Sex (male)	−1.050	1.689	38.126	−0.622	0.538
Baseline MoCA Score	−1.478	0.165	38.406	−8.966	5.89 × 10^−11^

**Table A3:** Fixed effects from the sensitivity analysis using the linear mixed-effects model with all available participants (*n* = 53), including those with only six months of follow-up.

	Estimate	SE	df	t Value	*p* Value
Intercept	44.349	7.052	45.125	6.289	1.15 × 10^−7^
Time (months)	0.191	0.037	199.660	5.126	6.97 × 10^−7^
Baseline Age (years)	0.053	0.092	46.063	0.571	0.571
Sex (male)	−0.031	1.656	44.257	−0.019	0.985
Baseline MoCA Score	−1.610	0.150	47.695	−10.723	2.66 × 10^−14^

**Table A4:** Fixed effects from the sensitivity analysis using the linear mixed-effects model restricted to participants with clinical diagnosis of AD (*n* = 36).

	Estimate	SE	df	t Value	*p* Value
Intercept	43.970	8.227	31.607	5.345	7.55 × 10^−6^
Time (months)	0.200	0.043	156.744	4.595	8.84 × 10^−6^
Baseline Age (years)	0.040	0.110	32.643	0.360	0.721
Sex (male)	−2.306	1.918	30.601	−1.203	0.238
Baseline MoCA Score	−1.447	0.186	32.472	−7.761	6.80 × 10^−9^

**Table A5:** Fixed effects from linear mixed-effects model examining the effect of AD medications (cholinesterase inhibitors or memantine) on ADAS-Cog trajectories.

	Estimate	SE	df	t Value	*p* Value
Intercept	35.191	7.416	36.133	4.745	3.24 × 10^−5^
Time (months)	0.181	0.066	155.117	2.754	6.58 × 10^−3^
AD Med. (yes)	3.075	1.963	63.229	1.566	0.122
Baseline Age (years)	0.111	0.093	36.665	1.189	0.242
Sex (male)	−0.266	1.692	35.729	−0.157	0.876
Baseline MoCA Score	−1.480	0.159	35.823	−9.335	3.96 × 10^−11^
Time: AD Med. (yes)	0.094	0.091	158.269	1.034	0.303

**Table A6:** Fixed effects from linear mixed-effects model examining the effect of antidepressant medications (SSRIs or other antidepressants) on ADAS-Cog trajectories.

	Estimate	SE	df	t Value	*p* Value
Intercept	40.238	7.186	35.326	5.599	2.53 × 10^−6^
Time (months)	0.230	0.051	158.638	4.527	1.17 × 10^−5^
Antidepressant (yes)	4.152	2.541	64.352	1.6234	0.107
Baseline Age (years)	0.064	0.094	36.514	0.681	0.500
Sex (male)	−1.211	1.700	35.024	−0.712	0.481
Baseline MoCA Score	−1.480	0.164	35.894	−9.052	8.53 × 10^−11^
Time: Antidepressant (yes)	−0.032	0.115	154.353	−0.277	0.782

**Table A7:** Fixed effects from linear mixed-effects model examining the effect of hypertension medications on ADAS-Cog trajectories.

	Estimate	SE	df	t Value	*p* Value
Intercept	42.345	7.369	35.935	5.746	1.53 × 10^−6^
Time (months)	0.272	0.068	155.957	4.022	8.97 × 10^−5^
Hypertension Med. (yes)	−1.875	1.998	63.188	−0.939	0.351
Baseline Age (years)	0.048	0.096	37.342	0.503	0.618
Sex (male)	−1.553	1.747	35.363	−0.889	0.380
Baseline MoCA Score	−1.441	0.167	36.349	−8.612	2.65 × 10^−10^
Time: Hypertension Med. (yes)	−0.078	0.091	155.828	−0.849	0.397

**Table A8:** Fixed effects from linear mixed-effects model examining the effect of cholesterol medications on ADAS-Cog trajectories.

	Estimate	SE	df	t Value	*p* Value
Intercept	40.599	7.531	35.015	5.391	4.90 × 10^−6^
Time (months)	0.274	0.061	157.476	4.518	1.22 × 10^−5^
Cholesterol Med. (yes)	0.442	2.064	60.516	0.214	0.831
Baseline Age (years)	0.066	0.101	36.036	0.657	0.515
Sex (male)	−1.361	1.777	35.74	−0.766	0.449
Baseline MoCA Score	−1.473	0.170	35.457	−8.643	2.99 × 10^−10^
Time: Cholesterol Med. (yes)	−0.111	0.092	156.304	−1.212	0.227
